# Identification of cancer-associated fibroblasts subtypes in prostate cancer

**DOI:** 10.3389/fimmu.2023.1133160

**Published:** 2023-03-24

**Authors:** Jiahua Pan, Zehua Ma, Bo Liu, Hongyang Qian, Xiaoguang Shao, Jiazhou Liu, Qi Wang, Wei Xue

**Affiliations:** ^1^ Department of Urology, Ren Ji Hospital, Shanghai Jiao Tong University School of Medicine, Shanghai, China; ^2^ Shanghai Key Laboratory for Tumor Microenvironment and Inflammation, School of Medicine, Shanghai Jiao Tong University, Shanghai, China

**Keywords:** cancer-associated fibroblasts, heterogeneity, castration resistance, prognosis, prostate cancer

## Abstract

**Introduction:**

Cancer-associated fibroblasts (CAFs) are one of the most abundant cell types in tumor microenvironment. However, the phenotypic and functional heterogeneities among CAFs have not been sufficiently investigated in prostate cancer.

**Methods:**

We obtained and analyzed the single-cell RNA-sequencing data from 26 hormone-sensitive prostate cancer samples and 8 castration-resistant prostate cancer samples, along with the analysis of bulk-sequencing datasets. Furthermore, we performed multicolor immunofluorescence staining to verify the findings from the data analysis.

**Results:**

We identified two major CAFs subtypes with distinct molecular characteristics and biological functions in prostate cancer microenvironment, namely αSMA+ CAV1+ CAFs-C0 and FN1+ FAP+ CAFs-C1. Another single-cell RNA-sequencing dataset containing 7 bone metastatic prostate cancer samples demonstrated that osteoblasts in the bone metastatic lesions comprised two subtypes with molecular characteristics and biological functions similar to CAFs-C0 and CAFs-C1 in the primary tumor sites. In addition, we discovered a transcriptional factor regulatory network depending on CAFs-C1. CAFs-C1, but not CAFs-C0, was associated with castration resistance and poor prognosis. We also found that CAFs-C1 signature was involved in treatment resistance to immune checkpoint inhibitors.

**Discussion:**

In summary, our results identified the presence of heterogeneous CAFs subtypes in prostate cancer microenvironment and the potential of specific CAFs subtype as therapeutic target for castration-resistant prostate cancer.

## 1 Introduction

Prostate cancer is a leading cause of male cancer-related death and remains the highest incidence worldwide (1, 2). Localized prostate cancer patients are typically treated with radical prostatectomy or radical radiotherapy (3). However, advanced prostate cancer inevitably develops into castration-resistant prostate cancer (CRPC) (4). Although recent developments in novel androgen receptor antagonists have significantly prolonged the survival of CRPC patients, acquired drug resistance still leads to tumor recurrence and metastasis (5, 6). Therefore, the discovery and development of novel therapeutic strategies for CRPC are highly desired.

In prostate cancer, efforts on exploring the molecular mechanisms underlying tumor progression and drug resistance have mainly focused on the tumor cell-intrinsic regulatory mechanisms, but increasing evidence have suggested that tumor microenvironment also plays an important role in tumor progression and drug resistance (7–10). Cancer-associated fibroblasts (CAFs) are one of the most abundant cell types in tumor microenvironment. Increasing studies have implicated that CAFs consist of heterogeneous subpopulations with diverse phenotypic and functional features. Meanwhile, specific CAFs subpopulation has protumoral effects in some tumor types (11–13). For instance, depletion of αSMA^+^ CAFs-secreted IL6 could improve the efficacy of gemcitabine in pancreatic ductal adenocarcinoma. FAP^+^ CAFs could recruit myeloid-derived suppressor cells through CCL2 to promote immunosuppression in intrahepatic cholangiocarcinoma (7, 14). Recently, a growing but limited number of studies have investigated the CAFs characteristics in prostate cancer microenvironment by single-cell RNA-sequencing (scRNA-seq) (15–22). The majority of these studies have focused more on exploring the heterogeneous tumor and immune cell subtypes in prostate cancer microenvironment, without thorough insights into the phenotypic and functional properties of CAFs subtypes. In addition, the most of these studies have not included the scRNA-seq data from CRPC samples, thus lacking sufficient information to investigate the dynamic evolution of CAFs subtypes during prostate cancer progression.

Here, we utilized scRNA-seq data from 26 HSPC and 8 CRPC samples as well as multicolor immunofluorescence staining to investigate the CAFs landscape in prostate cancer. We discovered two major CAFs subtypes in prostate cancer microenvironment, termed αSMA^+^ CAV1^+^ CAFs-C0 and FN1^+^ FAP^+^ CAFs-C1. The two CAFs subtypes had distinct molecular features and biological functions. STAT2 may be involved in the regulation of CAFs-C1 differentiation. Bulk-sequencing profiles suggested that CAFs-C1 signature, but not CAFs-C0 signature, was enriched in CRPC samples. High CAFs-C1 signature served as an adverse prognostic factor in prostate cancer, whereas CAFs-C0 signature had no association with castration resistance or patient prognosis. Moreover, we found the valuable ability of CAFs-C1 signature in predicting clinical outcomes and immunotherapy efficacy in some other tumor types. Overall, we identified two heterogeneous subpopulations of CAFs in prostate cancer microenvironment, which may provide insights into the discovery of potential novel therapeutic targets for CRPC.

## 2 Methods

### 2.1 scRNA-seq data collection and integration

We collected the scRNA-seq data for 26 hormonal-sensitive prostate cancer (HSPC) samples and 8 CRPC samples from 5 public datasets to study CAFs heterogeneity in prostate cancer (**Table S1**) (16–18, 23). First, we used Seurat V3 R package to import the raw counts data of each sample into R software (24). Next, we removed out cells from the raw counts data which met the following exclusion criteria: cells with less than 500 genes expressed, cells with more than 15% of genes derived from mitochondrial genes, or cells with more than 15% of genes derived from ribosomal genes. Since genes associated with mitochondria and ribosome can cause unexpected noise and such genes were not the focus of our study, we removed out the mitochondrial genes (genes beginning with MT-) and ribosomal genes (genes beginning with RPL or RPS) from the raw counts data. The DoubletFinder R package was utilized to remove the doublets from the raw counts data (25). In the remaining high-quality cells, Harmony R package was used to integrate multiple scRNA-seq datasets. After integration, the reduction was set as “harmony” for subsequent dimensionality reduction. We normalized and scaled the raw counts data using the NormalizeData and SclaeData function, respectively. To reduce dimensionality, we performed principal component analysis on the normalized data using the top 2000 highly variable features determined by the FindVariableFeatures function. The appropriate number of principal components was selected using the ElbowPlot function. The t-distributed stochastic neighbor embedding (t-SNE) and uniform manifold approximation and projection dimensionality reduction (UMAP) were then conducted by RunTSNE and RunUMAP function, respectively. The FindClusters function was implemented to identify potential cell clusters.

The scRNA-seq data for 7 bone metastatic prostate cancer samples and 8 normal bone samples was collected from GSE143791 (26). The processes of data quality control, multiple datasets integration, and standard data processing were consistent with the above.

### 2.2 Cluster markers identification

We annotated the cell clusters from the prostate cancer samples by the average expression of the following well-recognized cell type markers: T cells (PTPRC, CD3D, CD3E, and CD3G), B cells (CD79A, IGKC, IGLC2, and IGLC3), macrophages (CD68, CD163, FCGR3A, LYZ, and CSF1R), mast cells (ENPP3, KIT, SLC18A2, and MS4A2), fibroblasts (αSMA, FN1, and FAP), endothelial cells (PECAM1, ENG, CDH5, and VWF), and epithelial cells (EPCAM, AR, KRT5, KRT14, KRT8, and KRT18). For the annotation of cell clusters from bone metastatic prostate cancer samples, the cell type marker genes were obtained from Youmna et al. (26).

### 2.3 Differential gene expression analysis

The FindMarkers function embedded in Seurat V3 R package was used to identify the differentially expressed genes (DEGs) between different cell clusters or cell types. Genes that met the following criteria were defined as DEGs: genes expressed in at least 20% of cells, the adjusted P value ≤ 0.01, and the |Fold Change| ≥ 2. Function enrichment analysis of DEGs was performed using the Metascape online tool (http://metascape.org).

### 2.4 Trajectory analysis

We used monocle2 R package to investigate the cell lineage trajectory of CAFs (27). The dispersion Table function was used to identify significant genes that meet the criteria of mean_expression≥0.5 and dispersion_empirical≥1*dispersion_fit. Cells were then ordered using orderCells function. We used DDRTree function for reducing dimension and plot_cell_trajectory function for visualization. After the cell lineage trajectory was constructed, we used the differentialGeneTest function to identify DEGs along the pseudotime.

### 2.5 Calculation of signature score

We used gene set variation analysis function embedded in gsva R package to calculate the CAF-C0 signature score and CAF-C1 signature score (28). The detailed gene lists associated with the above scores were described in **Table S2**.

### 2.6 Regulons identification

We used pySCENIC to identify the potential gene regulatory networks in the scRNA-seq data (29). The regulons activities of transcriptional factors were calculated using the AUCell function embedded in pySCENIC. The analysis was performed with the default parameters. The input expression matrix was prefiltered using the following criteria: cells with more than 500 genes expressed and genes expressed in at least 5 cells.

### 2.7 Cell–cell interactions

To explore potential cell-cell interactions between cells, we used CellPhoneDB to identified significant receptor-ligand interactions (30). The analysis was performed with default parameters. Valid receptor-ligand interactions were defined as the expression of specific receptors in one cell type and the expression of the corresponding ligands in another cell type.

### 2.8 Bulk-transcriptomic data and analysis

The RNA-seq data and clinical information for TCGA-PRAD, TCGA-BLCA, TCGA-KIRC, TCGA-STAD, TCGA-LGG, TCGA-MESO, and TCGA-SKCM dataset were obtained from UCSC Xena (http://xena.ucsc.edu) (31). The gene expression matrix and clinical information for GSE116918 dataset were collected from the GEO database (32). The RNA-seq data and clinical information for metastatic urothelial carcinoma patients treated with anti-PD-L1 immunotherapy were obtained from http://research-pub.gene.com/IMvigor210CoreBiologies (33). The RNA-seq data and clinical information for metastatic melanoma patients treated with anti-PD1 immunotherapy were obtained from GEO database GSE78220 (34). The gene expression matrix for GSE32269, GSE2443, GSE6811, GSE31410, GSE21887, and GSE61379 was obtained from GEO database (35–40).

Gene Set Enrichment Analysis (GSEA) was applied using the GSEA software (Version: 3.0; http://software.broadinstitute.org/gsea/index.jsp). Significance was set at NES>1.0, P value<0.05, and FDR<0.25.

We performed hierarchical clustering on the normalized expression data of CAFs-C0 and CAFs-C1 signature components. Euclidean distance was chosen as the distance metric and Ward as the clustering method.

### 2.9 Immunohistochemistry and immunofluorescence staining

We collected 56 treatment-naive HSPC samples and 15 post-endocrine therapy CRPC samples from the Renji Hospital. Formalin-fixed and paraffin-embedded tumor tissues were collected. For antigen retrieval, 3 μm paraffin-embedded sections were unmasked in 1 x Tris-EDTA buffer for 20 minutes at 95°C. For immunohistochemistry staining, slides were incubated with αSMA (1:5000; Abcam; ab124964), FN1 (1:2500; Abcam; ab268020), or FAP (1:200; Abcam; ab207178) overnight at 4°C and then incubated with HRP-linked secondary antibody (1:500; Cell Signaling Technology; 7074S) for 1 hour at room temperature. DAB was used for visualization. Immunofluorescence staining was performed in 3 μm paraffin-embedded sections using the Three-color Fluorescence kit (Recordbio Biological Technology, Shanghai, China) based on the tyramide signal amplification technology according to the manufacture’s instruction. The antibody catalog numbers and staining concentrations used in immunofluorescence staining were: αSMA (1:5000; Abcam; ab124964), CAV1 (1:250; Cell Signaling Technology; 3238S), FN1 (1:2500; Abcam; ab268020), FAP (1:200; Abcam; ab207178), and STAT2 (1:200; Abcam; ab32367).

### 2.10 Statistical analysis

All statistics and graphs were completed using the R software (v3.6.3) and GraphPad Prism software (v8.0). We used two-tailed t-test or Wilcoxon rank-sum test to calculated the P value. Kaplan–Meier survival analyses were completed using survival and survminer R packages. Data were represented as mean values ± SD. Significant P value was set at <0.05.

## 3 Results

### 3.1 Identification of CAFs subtypes in prostate cancer microenvironment

**Figure 1:**
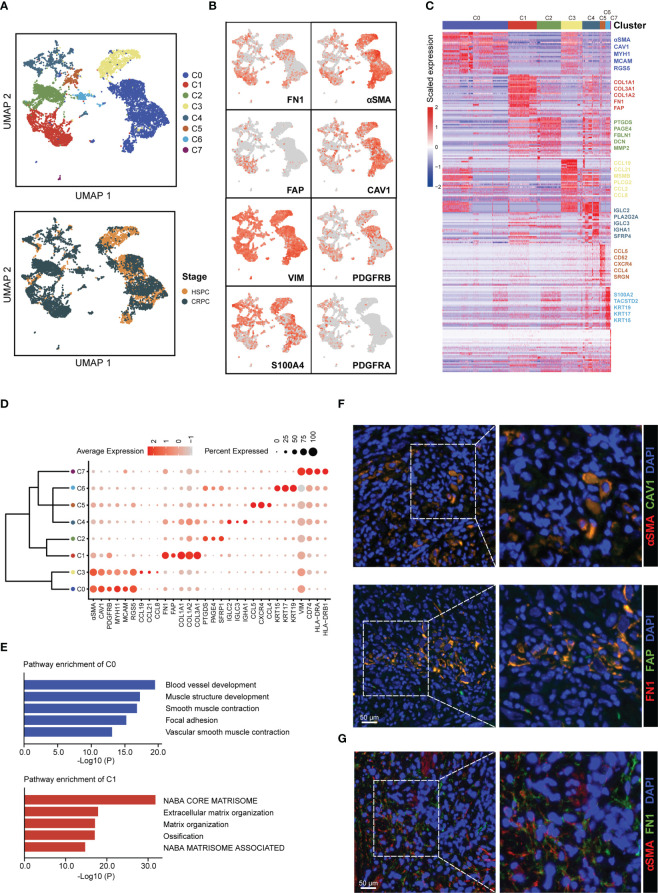
Identification of CAFs subtypes in prostate cancer microenvironment. **(A)** UMAP plot of CAFs colored by subtypes (top) and tumor stages (bottom), with 4,584 fibroblasts from HSPC samples and 3,732 fibroblasts from CRPC samples. **(B)** UMAP plot showing the expression levels of fibroblasts marker genes. **(C)** Heatmap showing the DEGs between different CAFs subtypes. **(D)** Dot plot displaying the expression levels of representative CAFs subtype-marker genes. **(E)** Top 5 enriched pathways for CAFs-C0 and CAFs-C1. **(F)** Representative fluorescence images of αSMA^+^ CAV1^+^ (top) and FN1^+^ FAP^+^ CAFs (bottom) from prostate cancer samples. Scale bar, 50 μm. **(G)** Representative fluorescence image of αSMA and FAP-stained prostate cancer samples. Scale bar, 50 μm.

To identify CAFs subtypes in prostate cancer microenvironment, we collected scRNA-seq data for 26 HSPC samples and 8 CRPC from 5 public datasets (**Table S1**). After data quality control, multiple datasets integration, and standard data processing, we discovered seven cell types in the prostate cancer microenvironment, including epithelial cells, T cells, B cells, macrophages, mast cells, fibroblasts, and endothelial cells (**Figures S1A–C**). Reclustering of 8,316 fibroblasts generated eight CAFs subtypes (**Figure 1A**). We next assessed the expression of well-established fibroblasts marker genes among the eight subtypes (C0-C7). Several genes (VIM and S100A4) were highly expressed in all eight CAFs subtypes, whereas other genes (FN1, FAP, αSMA, CAV1, PDGFRA, and PDGFRB) were nonuniformly expressed among the eight CAFs subtypes (**Figure 1B**). As shown in **Figures 1C, D**, these eight CAFs subtypes could be distinguished from each other by the expression of subtype-specific marker genes. Specially, CAFs-C0 highly expressed αSMA, CAV1, and microvasculature signature genes such as MYH1, MCAM, and RGS5, while CAFs-C1 highly expressed FN1, FAP, and collagen molecules such as COL1A1, COL3A1, and COL1A2. Pathway enrichment analysis on specific marker genes of each CAFs subtype revealed the functional heterogeneities among CAFs subtypes (**Figures 1E**, **S1D**). Pathways related to microvasculature development were enriched in CAFs-C0, while CAFs-C1 had high levels of extracellular matrix organization signatures. Like CAFs-C1, the path-ways enriched in CAFs-C2 were also related to extracellular matrix organization. CAFs-C3 had significant enrichment of inflammatory response regulation signatures. Since scRNA-seq analysis revealed that αSMA^+^ CAV1^+^ and FN1^+^ FAP^+^ CAFs were the two most abundant subpopulations of CAFs in prostate cancer microenvironment, we thus stained prostate cancer samples with these four fibroblasts markers to verified our findings from the bioinformatic analysis. The staining results confirmed the co-expression of αSMA and CAV1 and the co-expression of FN1 and FAP (**Figure 1F**), with minimal overlap between αSMA and FN1 expression (**Figure 1G**). Recent scRNA-seq analysis of different tumors have revealed various CAF subtypes, notably myofibroblastic CAFs (myCAFs) and inflammatory CAFs (iCAFs). myCAFs generally highly express collagen molecules and αSMA has been widely recognized as the marker for myCAFs (41–44). However, our analysis revealed that FN1^+^ FAP^+^ CAFs-C1, but not αSMA^+^ CAV1^+^ CAFs-C0, had high expression levels of collagen molecules and contributed to extracellular matrix remodeling. Of note, while some studies, including our analysis, have revealed that αSMA^+^ and FAP^+^ CAFs are distinct subpopulations of CAFs (7, 42), other studies have reported the co-expression of αSMA and FAP on CAFs, which suggests the considerable heterogeneity among tumors (43, 45, 46). iCAFs, characterized by cytokine secretion, have been reported to be involved in tumor progression and treatment resistance (8, 11), but our analysis showed that the two most abundant CAFs subtypes in prostate cancer microenvironment did not have the feature of cytokine secretion.

### 3.2 Compositional and transcriptional profile changes associated with CAFs subtypes

**Figure 2:**
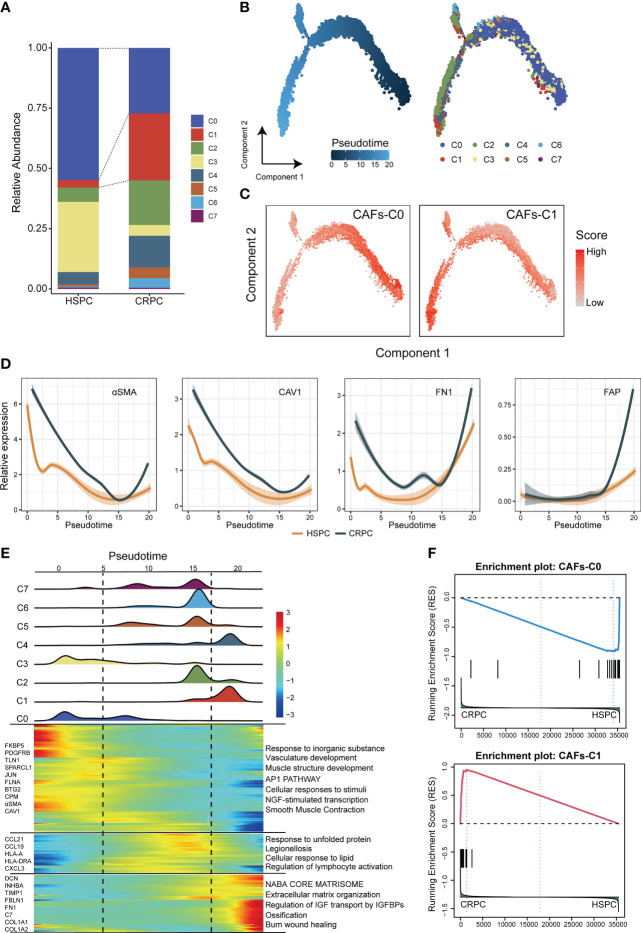
Compositional and transcriptional profile changes associated with CAFs subtypes. **(A)** Relative abundance of each CAFs subtype in HSPC and CRPC samples. **(B)** Trajectory analysis of CAFs inferred by Monocle2. **(C)** The dynamics of CAFs-C0 and CAFs-C1 signature scores along the pseudotime. **(D)** The dynamic expression of CAFs-C0 marker genes (αSMA and CAV1) and CAFs-C1 marker genes (FN1 and FAP) along the pseudotime. **(E)** Bottom. heatmap displaying the dynamics in gene expression along the pseudotime. Top. the distribution of CAFs subtypes along the pseudo-time. **(F)** GSEA analysis for CAFs-C0 and CAFs-C1 signature between HSPC-derived versus CRPC-derived CAFs.

The subtype composition of CAFs changed with disease progression: CAFs-C0 as the most abundant subtype in HSPC but CAFs-C1 as the most abundant subtype in CRPC (**Figure 2A**). To further investigate the dynamic transition of compositional and transcriptional profiles in CAFs during disease progression, we performed trajectory analysis of CAFs using Monocle2. Trajectory analysis revealed that CAFs-C0 were at the early stage of the trajectory path, whereas CAFs-C1 were at the late stage of the trajectory path (**Figure 2B**). We then analyzed the dynamics of CAFs-C0 and CAFs-C1 signature scores along the pseudotime. We defined these signatures by obtaining the overlapping genes between upregulated DEGs of fibroblasts and upregulated DEGs of CAFs-C0 or CAFs-C1 (**Figures S2A, B**; **Tables S2, S3**), only retaining genes specific for both fibroblasts and CAFs subtypes. Consistently, CAFs-C0 signature scores were downregulated, while CAFs-C1 signature scores were upregulated along the pseudo-time (**Figure 2C**). We also observed significant downregulation of CAFs-C0 marker genes (αSMA and CAV1) but significant upregulation of CAFs-C1 marker genes (FN1 and FAP) during these transitions (**Figure 2D**). We next analyzed the transcriptional profile changes associated with CAFs state transitions and divided the transcriptional profile changes into 3 phases (**Figure 2E**). CAFs-C0 and CAFs-C3 were phase 1 cells, CAFs-C1 and CAFs-C4 were phase 3 cells, and other CAFs subtypes were phase 2 cells (**Figure 2E**). Phase 1 cells had high expression levels of αSMA, CAV1, PDGFRB, TLN1, and FLNA, matching the specific marker genes of CAFs-C0 and CAFs-C3 (**Figure 2E**; **Table S2**). Phase 3 cells highly expressed extracellular matrix markers such as DCN, FBLN1, FN1, COL1A1, and COL1A2, matching the specific marker genes of CAFs-C1 (**Figure 2E**; **Table S2**). Pathway analysis suggested that phase 1 cells were implicated in vasculature development, whereas phase 3 cells were involved in extra-cellular matrix remodeling. These results indicated that CAFs had different properties along the trajectory path, with a CAFs-C0 phenotype in the early stage but a CAFs-C1 phenotype in the late stage. Furthermore, GSEA analysis reveals that CAF-C0 signature was enriched in HSPC samples and CAF-C1 signature was enriched in CRPC samples (**Figure 2F**).

### 3.3 Gene regulatory programs and cell-cell communication networks associated with CAFs subtypes

**Figure 3:**
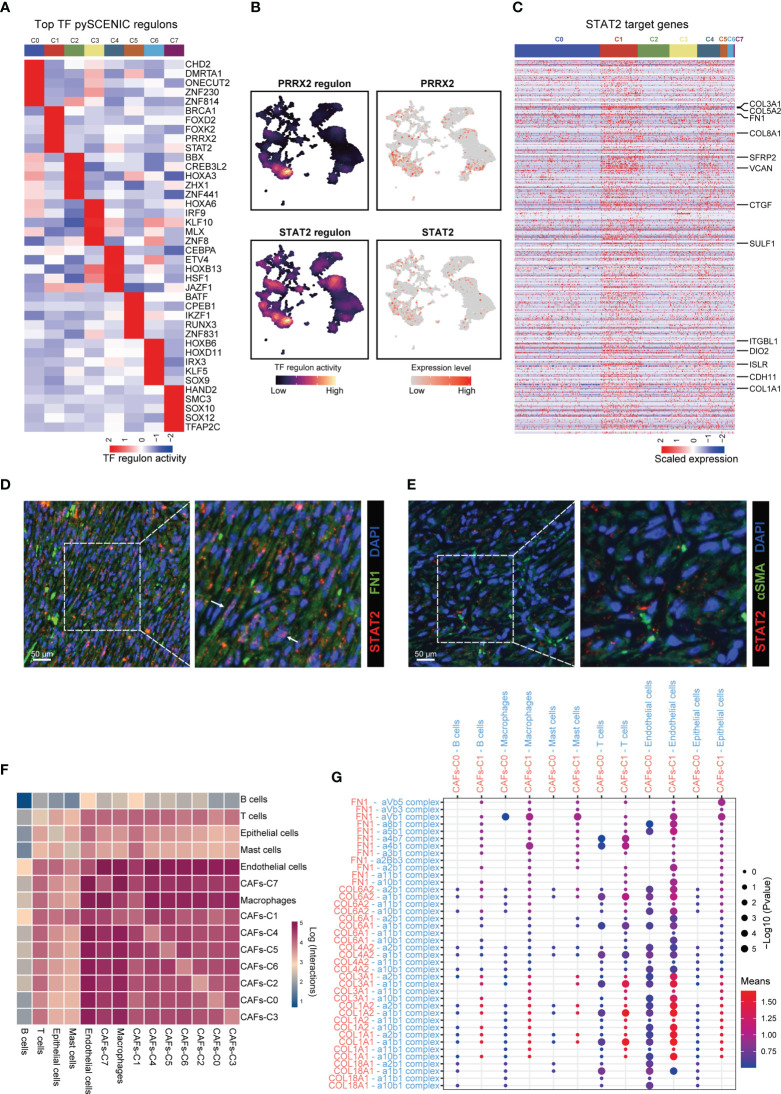
Gene regulatory programs and cell-cell communication networks associated with CAFs subtypes. **(A)** Heatmap showing the regulon activities of top 5 activating transcriptional factors for each CAFs subtype predicted by pySCENIC. **(B)** UMAP plot showing the PRRX2- and STAT2-regulon activity predicted by pySCENIC. **(C)** Heatmap showing the expression levels of STAT2 target genes in each CAFs subtype. Upregulated DEGs of CAFs-C1 were labeled. **(D)** Representative fluorescence images of STAT2^+^ FN1^+^ CAFs from prostate cancer samples. Scale bar, 50 μm. **(E)** Representative fluorescence images of STAT2 and αSMA-stained prostate cancer samples. Scale bar, 50 μm. **(F)** Heatmap showing the number of potential cell-cell interactions between CAFs and all other cell types predicted by CellPhoneDB. **(G)** Dot plot of representative cell-cell interactions between CAFs-C0, CAFs-C1, and all other cell types.

To investigate the regulatory programs which may lead to the substantial differences in transcriptional profile among CAFs subtypes, we investigated the transcriptional regulatory networks for CAFs. pySCENIC inferred the top five activating transcriptional factors for each CAFs subtype (**Figure 3A**). Among these transcriptional factors, both STAT2 and PRRX2 had high expression levels and transcription activities in CAFs-C1 (**Figures 3A, B**, **S3A**). However, only the downstream target genes of STAT2 contained the specific marker genes for CAFs-C1 (**Figures 3C**, **S3B**). Therefore, STAT2, but not PRRX2, may be involved in the regulation of CAFs-C1 differentiation. To exclude the influences of other STAT family members in CAFs-C1, we examined the expression levels and transcription activities of other STAT family members in CAFs-C1. The expression levels and transcription activities of other STAT family members were considerably lower than those of STAT2 in CAFs-C1 (**Figures S3C, D**). Subsequently, we evaluated the relationship between STAT2 expression and CAFs subtypes by immunofluorescence staining. The staining results suggested that CAFs-C1 exhibited high expression of STAT2, whereas CAFs-C0 lacked expression of STAT2 (**Figures 3D, E**). Additionally, by mining the prostate cancer dataset from TCGA, we observed a significant positive correlation between the expression levels of STAT2 and CAFs-C1 marker FN1, while no significant correlation was observed between the expression levels of STAT2 and CAFs-C0 marker αSMA (**Figure S4**). These results suggested that STAT2 may participated in the differentiation of CAFs-C1.

Using CellPhoneDB, we investigated the cell–cell communication networks between CAFs and other cell types. CAFs showed frequent interactions with other cell types and endothelial cells were the predominant cell type interacting with CAFs (**Figure 3F**). We next focused on the cell–cell interactions between CAFs-C0, CAFs-C1, and other cell types. The most frequent cell-cell interactions between CAFs-C0, CAFs-C1, and other cell types were related to extracellular matrix organization (**Figure 3G**).

### 3.4 Identification of osteoblasts subtypes in bone metastatic lesions

**Figure 4:**
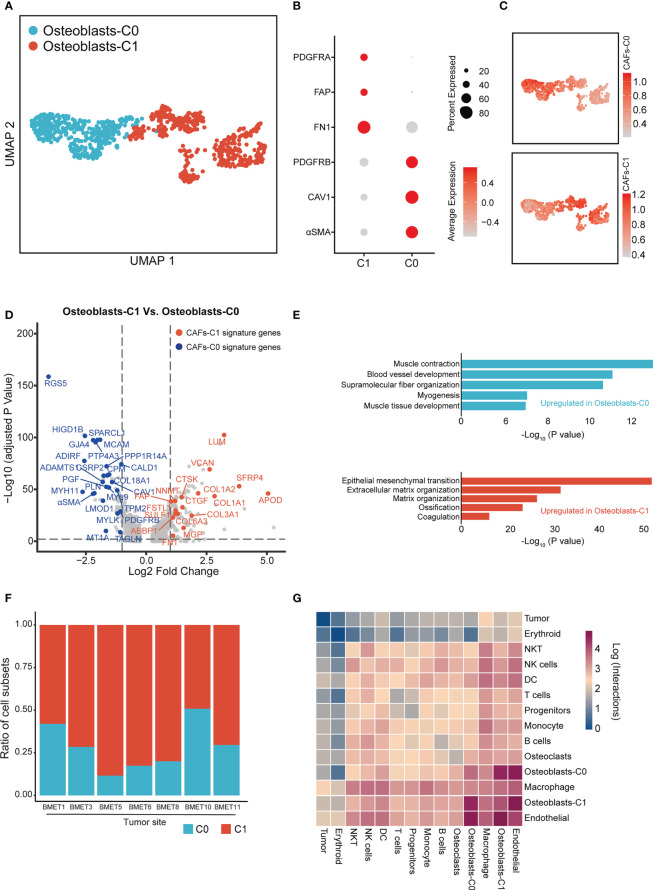
Identification of osteoblasts subtypes in bone metastatic lesions. **(A)** UMAP plot of osteoblasts in 7 bone metastatic prostate cancer samples. Osteoblasts subtypes were demarcated by colors. **(B)** Dot plot showing the expression levels of representative CAFs-C0 and CAFs-C1 signature genes in each osteoblasts subtype. **(C)** UMAP plot showing the CAFs-C0 and CAFs-C1 signature scores in osteoblasts. **(D)** Volcano plot displaying the DEGs between osteoblasts-C1 versus osteoblasts-C0. CAFs-C0 and CAFs-C1 signature genes were represented in blue and red, respectively. **(E)** Top 5 enriched pathways for osteoblasts-C0 and osteoblasts-C1. **(F)** Relative abundance of each osteoblasts subtype in 7 bone metastatic prostate cancer samples. **(G)** Heatmap showing the number of potential cell-cell interactions between osteoblasts and all other cell subtypes predicted by CellPhoneDB.

Bone metastases are the primary cause of mortality in prostate cancer patients (47–49). Osteoblasts are responsible for the formation of bone matrix by secreting and organizing collagens and other proteins. A large body of evidence implies that prostate cancer cells frequently interact with osteoblasts in the bone metastatic microenvironment, which are crucial for the metastatic colonization and proliferation of prostate cancer cells (50, 51). To investigate the osteoblasts landscape in the bone metastatic microenvironment, we collected scRNA-seq data for 7 bone metastatic prostate cancer samples. Thirteen major cell types were identified in the bone meta-static microenvironment (**Figures S5A–C**). Reclustering of osteoblasts produced two subtypes, termed osteoblasts-C0 and osteoblasts-C1 subtype (**Figure 4A**). Osteoblasts-C0 and osteoblasts-C1 highly expressed specific marker genes of CAFs-C0 and CAFs-C1, respectively (**Figure 4B**). CAFs-C0 signature and CAFs-C1 signature scores exhibited non-uniform distribution among osteoblasts (**Figure 4C**). We next sought to identify the DEGs between osteoblasts-C1 and osteoblasts-C0 (**Figure 4D**). The upregulated genes of osteoblasts-C0 contained lots of CAFs-C0 signature genes, while osteoblasts-C1 was characterized by increased expression of CAFs-C1 signature genes. Pathway analysis on the DEGs of osteoblasts-C0 and osteoblasts-C1 showed that osteoblasts-C0 were related to blood vessel development and osteoblasts-C1 were involved in extracellular matrix remodeling (**Figure 4E**). Additionally, the osteoblasts-C1 was the most abundant subtype of osteoblasts in the bone metastatic microenvironment (**Figure 4F**). We also analyzed the scRNA-seq data for 8 normal bone samples and found that the distribution of osteoblasts was absent in normal bone samples (**Figure S6**). These results revealed that osteoblasts in the bone metastatic lesions and CAFs in the primary tumor sites demonstrated similar phenotypic and functional properties, which suggested that CAFs-C0 and CAFs-C1 signatures were the two most predominant phenotypic signatures of CAFs and osteoblasts in prostate cancer. We also sought to investigated the cell–cell interaction networks between osteoblasts and other cell types. In the bone metastatic microenvironment, endothelial cells were the predominant cell type interacting with osteoblasts (**Figure 4G**), consistent with our earlier observations in the primary tumor microenvironment.

### 3.5 CAFs subtype signatures in bulk-sequencing profiles

**Figure 5:**
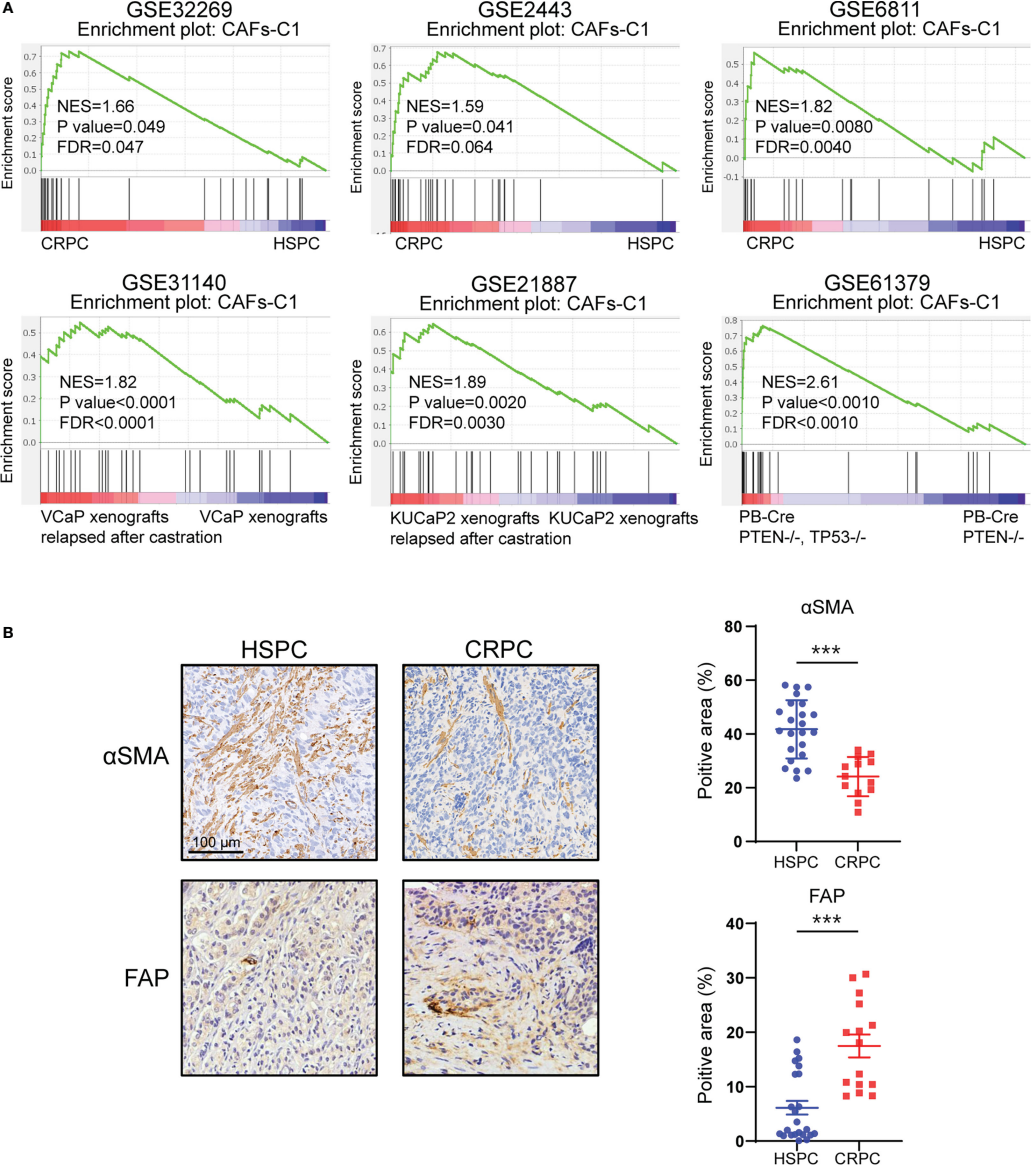
CAFs subtype signatures in bulk-sequencing profiles. **(A)** GSEA analysis for CAFs-C1 signature between HSPC versus CRPC samples. **(B)** Representative immunohistochemistry images and quantifications of αSMA and FAP in HSPC and CRPC sample sections. Scale bars, 100 μm. Data shown as mean ± SD. ***P<0.001.

To further explore the role of CAFs heterogeneity in CRPC development, we extended our analysis to bulk-sequencing profiles. GSEA analyses revealed significant enrichment of CAFs-C1 signature in CRPC samples compared with HSPC samples (**Figure 5A**). However, there was no difference in enrichment of CAFs-C0 signature between HSPC and CRPC samples (**Figure S7A**). We next examined the protein expression of αSMA and FAP in HSPC and CRPC samples. Immunohistochemistry staining suggested that CRPC samples exhibited decreased αSMA expression but increased FAP expression in comparison to HSPC samples (**Figure 5B**).

### 3.6 Prognostic significance of CAFs subtype signatures in prostate cancer

**Figure 6:**
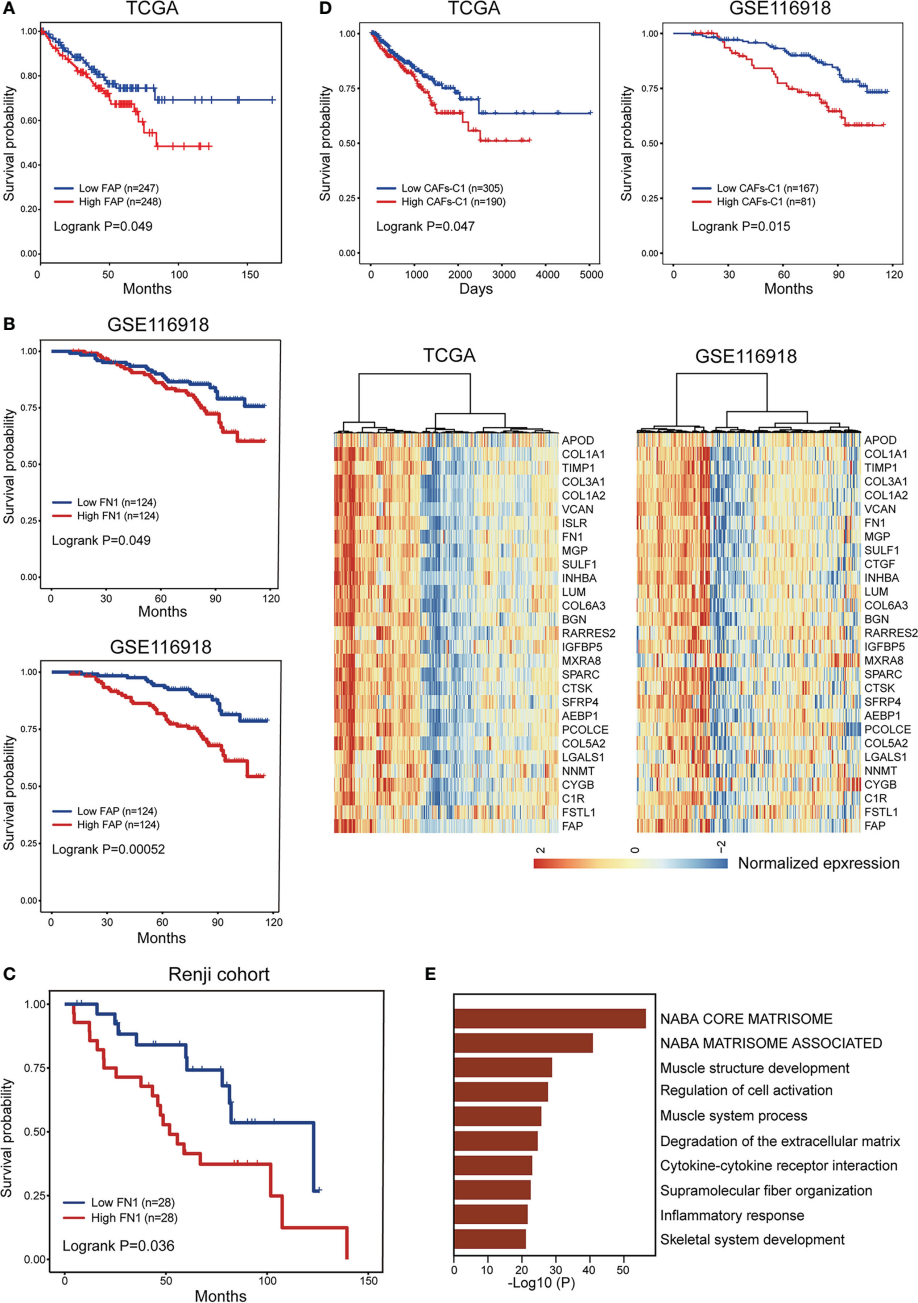
Prognostic significance of CAFs subtype signatures in prostate cancer. **(A)** The comparison of progression-free survival between patient subgroups divided by the expression level of FAP in TCGA-PRAD dataset. **(B)** The comparison of biochemical recurrence-free survival between patient subgroups divided by the expression levels of FN1 and FAP in GSE116918 dataset. **(C)** The comparison of biochemical recurrence-free survival between patient subgroups divided by the expression level of FN1 in Renji cohort. **(D)** Bottom. Hierarchical clustering defined two CAFs-C1 subtypes based on the normalized expression profiles of 30 CAFs-C1 signature genes in TCGA-PRAD and GSE116918 dataset. Top. Kaplan-Meier analysis showed high CAFs-C1 represented an adverse prognostic factor in TCGA-PRAD and GSE116918 dataset. **(E)** The top ten enriched pathways for high CAFs-C1 samples from **(D)**.

Previous studies indicate that different CAFs subtypes can be associated with different clinical prognosis, we therefore evaluated the prognostic significance of heterogeneous CAFs subtypes in prostate cancer. The expression levels of αSMA and CAV1 were not correlated with patient prognosis in TCGA-PRAD cohort (**Figure S8A**). High CAV1 expression was correlated with favorable prognosis in GSE116918 cohort (**Figure S8B**). In contrast, high expression of FN1 and FAP was associated with poor prognosis in both TCGA-PRAD and GSE116918 cohort (**Figures 6A, B**). Our data consistently showed that expression of αSMA had no association with patient prognosis while high FN1 expression was associated with poor prognosis in Renji cohort (**Figures 6C**, **S8C**). To better evaluated the prognostic significance of heterogeneous CAFs subtypes, we classified prostate cancer patients into different subtypes by hierarchical clustering of normalized expression profiles of CAFs-C0 and CAFs-C1 signature genes. Survival analyses suggested that patients with higher CAFs-C1 signature had worse prognosis, whereas CAFs-C0 signature had no association with patient prognosis in TCGA-PRAD and GSE116918 cohort (**Figures 6D**, **S8D**). Of note, pathway analysis on the DEGs of high CAFs-C1 patients from TCGA-PRAD cohort revealed significant enrichments of pathways related to extracellular matrix remodeling (**Figure 6E**).

### 3.7 Clinical significance of CAFs-C1 signature in distinct cancer types

**Figure 7:**
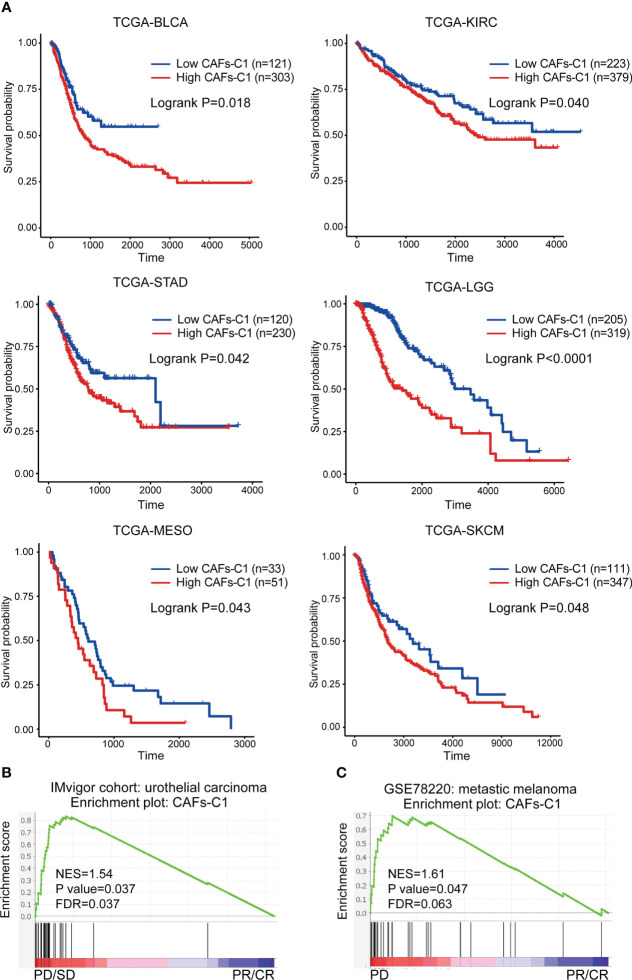
Clinical significance of CAFs-C1 signature in distinct cancer types. **(A)** Kaplan-Meier analysis comparing the overall survival differences among patients with high and low CAFs-C1 signature in TCGA-BLCA, TCGA-KIRC, TCGA-STAD, TCGA-LGG, TCGA-MESO, and TCGA-SKCM datasets. **(B)** GSEA analysis for meta-static urothelial carcinoma patients treated with anti-PD-L1 showing significant enrichment of CAFs-C1 signature in the PD and SD patients compared to CR and PR patients. **(C)** GSEA analysis for metastatic melanoma cancer patients treated with anti-PD1 showing significant enrichment of CAFs-C1 signature in the PD patients compared to CR and PR patients.

Since we identified that CAFs-C1 signature enrichment was an adverse prognostic factor in prostate cancer, we sought to investigate the clinical significance of CAFs-C1 signature in distinct cancer types. As shown in **Figure 7A**, high CAFs-C1 signature was associated with poor overall survival in bladder cancer (TCGA-BLCA), kidney renal clear cell carcinoma (TCGA-KIRC), stomach adenocarcinoma (TCGA-STAD), brain lower grade glioma (TCGA-LGG), mesothelioma (TCGA-MESO), and skin cutaneous melanoma (TCGA-SKCM). Specific subpopulation of CAFs could suppress antitumor immunity by secreting inflammatory cytokines and recruiting immunosuppressive cells (14, 52, 53), we thus studied whether CAFs-C1 signature was involved in treatment resistance to immunotherapy. To this end, we acquired two datasets which contained the transcriptional profiles and treatment information of urothelial carcinoma patients receiving anti-PD-L1 immunotherapy and melanoma patients receiving anti-PD1 immunotherapy. GSEA analyses showed that non-responders had significant enrichment of CAFs-C1 signature in comparison to responders, indicating that CAFs-C1 signature was involved in treatment resistance to immune checkpoint inhibitors **Figures 7B, C**.

## 4 Discussion

Numerous studies suggest that CAFs comprise several heterogeneous subsets of cells, yet the specific molecular features and biological functions of CAFs subsets during tumor formation and progression remain poorly understood and vary greatly across tumor types. Moreover, increasing studies demonstrate that only specific subsets of CAFs participate in tumor progression and targeting pan-CAFs leads to acceleration of some tumors (41, 54, 55). Therefore, identification of tumor-promoting subsets of CAFs is essential for CAFs-targeted therapeutic strategy. As the development of scRNA-seq technology, great progress has been made in identifying the specific markers and functions of different CAFs subsets in many tumor types. IL-6 secreted by vascular CAFs could promote tumor cell stemness in intrahepatic cholangiocarcinoma (56). Inflammatory CAFs senescence induced by irradiation confers chemoradiotherapy resistance to rectal cancer (8, 56). Recent studies identified αSMA^+^ and FAP^+^ CAFs with opposing functions in pancreatic ductal adenocarcinoma. αSMA^+^ CAFs could restrain tumor progression *via* producing type I collagen and depletion of αSMA^+^ CAFs leads to more aggressive tumor and impairs survival (41). In contrast, FAP^+^ CAFs could promote tumor progression and depletion of FAP^+^ CAFs results in tumor suppression and improved survival (7).

In this study, we identified eight CAFs subtypes in prostate cancer microenvironment, revealing that the CAFs landscape is highly heterogeneous. αSMA^+^ CAV1^+^ CAFs-C0 and FN1^+^ FAP^+^ CAFs-C1 were the two most prevalent CAFs subpopulation. The two CAFs subtypes had distinct roles in prostate cancer development. CAFs-C0 highly expressed microvasculature signature genes such as MYH1, MCAM, and RGS5, indicating that this CAFs subtype was related to microvasculature development. CAFs-C1 was characterized by increased expression of collagen molecules such as type I collagen and DCN, suggesting that this CAFs subtype was involved in extracellular matrix remodeling. αSMA^+^ CAV1^+^ CAFs-C0 and FN1^+^ FAP^+^ CAFs-C1 were predominantly enriched in HSPC and CRPC samples, respectively. Importantly, CAFs-C1, but not CAFs-C0, was associated with castration resistance and poor prognosis. Moreover, we found that osteoblasts in the bone metastatic lesions comprised two subtypes with molecular characteristics and biological functions similar to CAFs-C0 and CAFs-C1. These results were supported by scRNA-seq analyses, bulk-sequencing analyses, and tumor specimen staining.

Growing evidence supports that CAFs can originate from different cell populations and switch into different phenotypes in response to different stimuli, which lead to the phenotypical and functional heterogeneity among CAFs (8, 57). We found that STAT2 was strongly activated in CAFs-C1 and the target genes for STAT2 contained lots of specific marker genes of CAFs-C1. These results implied that STAT2 may be the key transcription factor driving the CAFs-C1 phenotype transformation. Of note, the mechanisms driving CAFs phenotype transformation are multifactorial and complicated, future studies are needed to further investigate the underlying mechanisms. Moreover, since osteoblasts and CAFs exhibited similar phenotypic and functional properties, there might be specific stimuli promoting the CAFs-C0 and CAFs-C1 phenotype transformation in the primary and metastatic lesions. Tumor initiation and progression are determined by both the intrinsic properties of tumor cells and extrinsic influences from tumor microenvironment. As critical components of tumor microenvironment, CAFs can not only interact with tumor cells but also other components of tumor microenvironment. CAFs can promote intratumoral T cells suppression by activating the immune checkpoints on the T cells surface (53). Extracellular vesicles secreted by CAFs contributed to drug resistance of gastric cancer cells *via* activating FAK-YAP signaling (58). Using CellPhoneDB, we identified that endothelial cells were the predominant cell type interacting with CAFs in the prostate cancer microenvironment, and most of the cell-cell interactions between CAFs and other cell types were involved in extracellular matrix organization. However, cytokines, exosomes, and extracellular vesicles secreted by CAFs may also influence the development of prostate cancer, which cannot be identified by CellPhoneDB analysis.

In conclusion, this study demonstrates that CAFs are heterogeneous in molecular features and biological functions, and different CAFs subtypes have different clinical significance and therapeutic implications for prostate cancer.

## Data availability statement

The datasets presented in this study can be found in online repositories. The names of the repository/repositories and accession number(s) can be found in the article/**Supplementary Material**.

## Ethics statement

Written informed consent was obtained from the individual(s) for the publication of any potentially identifiable images or data included in this article.

## Author contributions

JP, QW, and WX contributed to conception and design of the study. JP, ZM, BL, and HQ organized the database. JP, ZM, and BL performed the statistical analysis. JP, ZM, and BL wrote the first draft of the manuscript. HQ, XS, and JL wrote sections of the manuscript. QW and WX obtained the funding. All authors contributed to manuscript revision, read, and approved the submitted version.
