# Supplementary material for: Identification of cancer-associated fibroblasts subtypes in prostate cancer
Source: Front Immunol. 2023 Mar 24;14:1133160. doi: 10.3389/fimmu.2023.1133160 (PMC10080037; doi:10.3389/fimmu.2023.1133160)
Supplement: Supplementary file 1 [file Image_1.pdf]

Supplementary Material

Identification of cancer-associated fibroblasts subtypes in prostate cancer

Jiahua Pan 1†, Zehua Ma 1†, Bo Liu 1†, Hongyang Qian 1†, Xiaoguang Shao 1, Jiazhou Liu 1, Qi Wang 1,2*, and Wei Xue 1*

1Department of Urology, Ren Ji Hospital, Shanghai Jiao Tong University School of Medicine, Shanghai 200120, China.
2Shanghai Key Laboratory for Tumor Microenvironment and Inflammation, School of Medicine, Shanghai Jiao Tong University, Shanghai 200120, China.
†These authors contributed equally to this work and share first authorship.

* Correspondence:
Qi Wang, Department of Urology, Ren Ji Hospital, Shanghai Jiao Tong University School of Medicine, Shanghai 200120, China; Phone: 86-21-63846590; Fax: 86-21-58394262; E-mail: wqi@sjtu.edu.cn.
Wei Xue, Department of Urology, Ren Ji Hospital, Shanghai Jiao Tong University School of Medicine, Shanghai 20012. China; Phone: 86-21-63846590; Fax: 86-21-58394262; E-mail: xuewei@renji.com.

Supplementary Figures and Tables

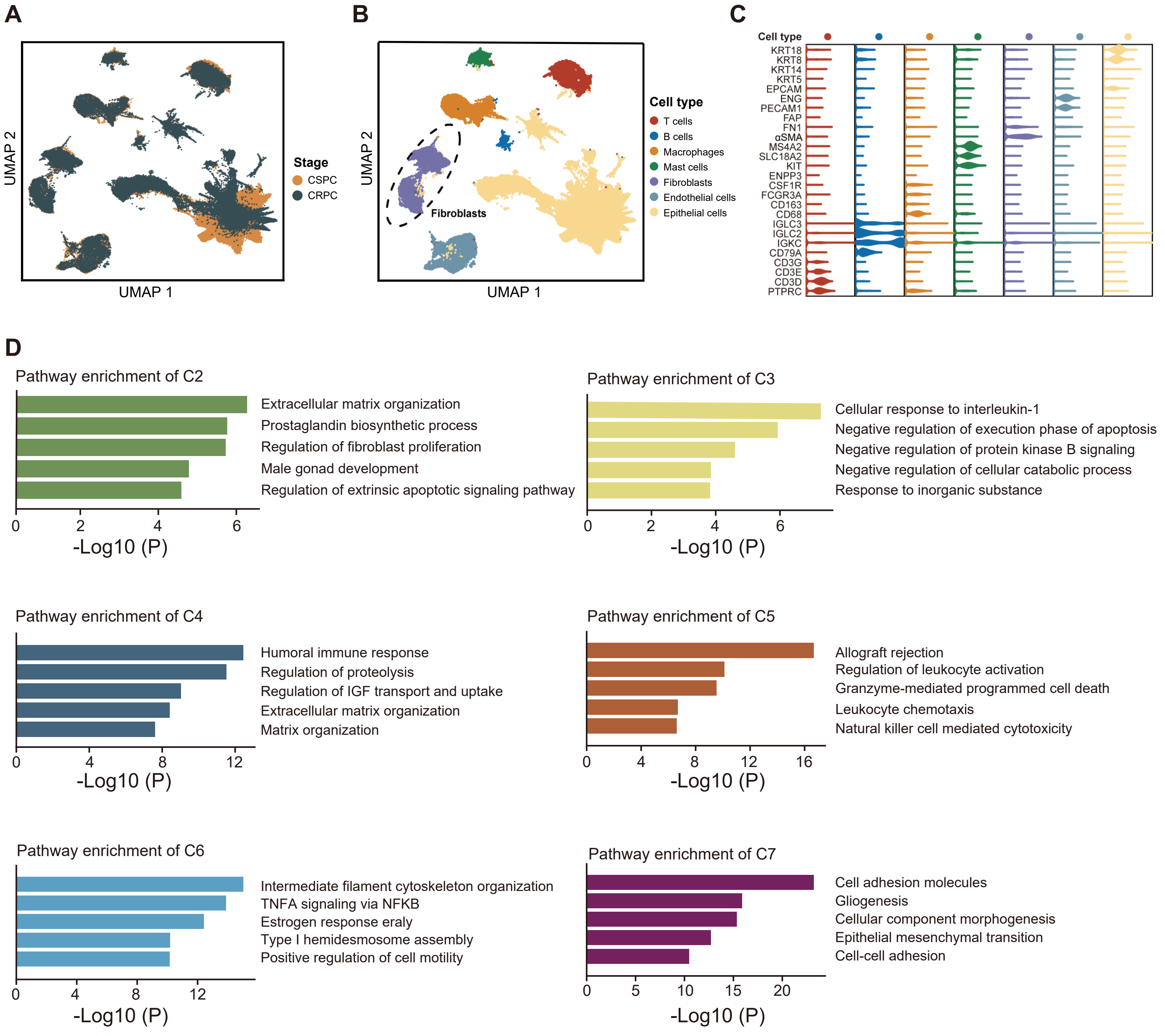

Supplemental figure 1. ScRNA-seq analysis of prostate cancer samples. A. UMAP plot showing the distribution of single cells across tumor stages. **B.** UMAP plot of single cells in 26 CSPC and 8 CRPC samples; cell types were annotated by colors.**C.** Violin plot displaying the expression levels of cell type marker genes for each cell type. **D.** Top 5 enriched pathways for each CAFs subtype (C2-C7).

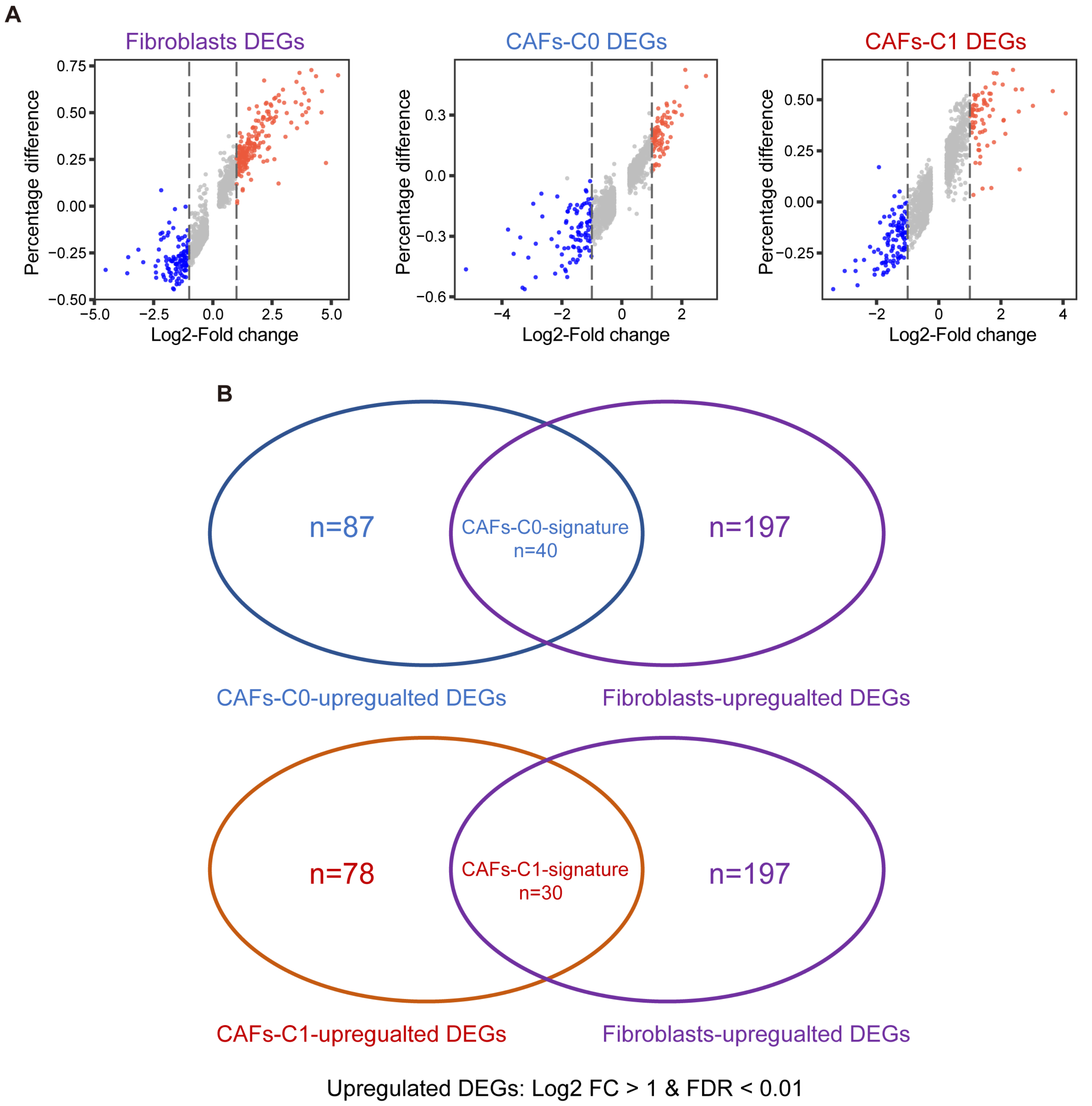

Supplemental figure 2. Identification of gene signature sets for CAFs subtypes. A. Volcano plots showing DEGs (absolute Log2 FC > 1; adjusted P < 0.01) in fibroblasts (versus other cell types), CAFs-C0 (versus other CAFs subtypes), and CAFs-C1 (versus other CAFs subtypes). **B.** Venn diagram showing the overlap between fibroblasts upregulated DEGs and CAFs-C0 upregulated DEGs (top) or CAFs-C1 upregulated DEGs (bottom).

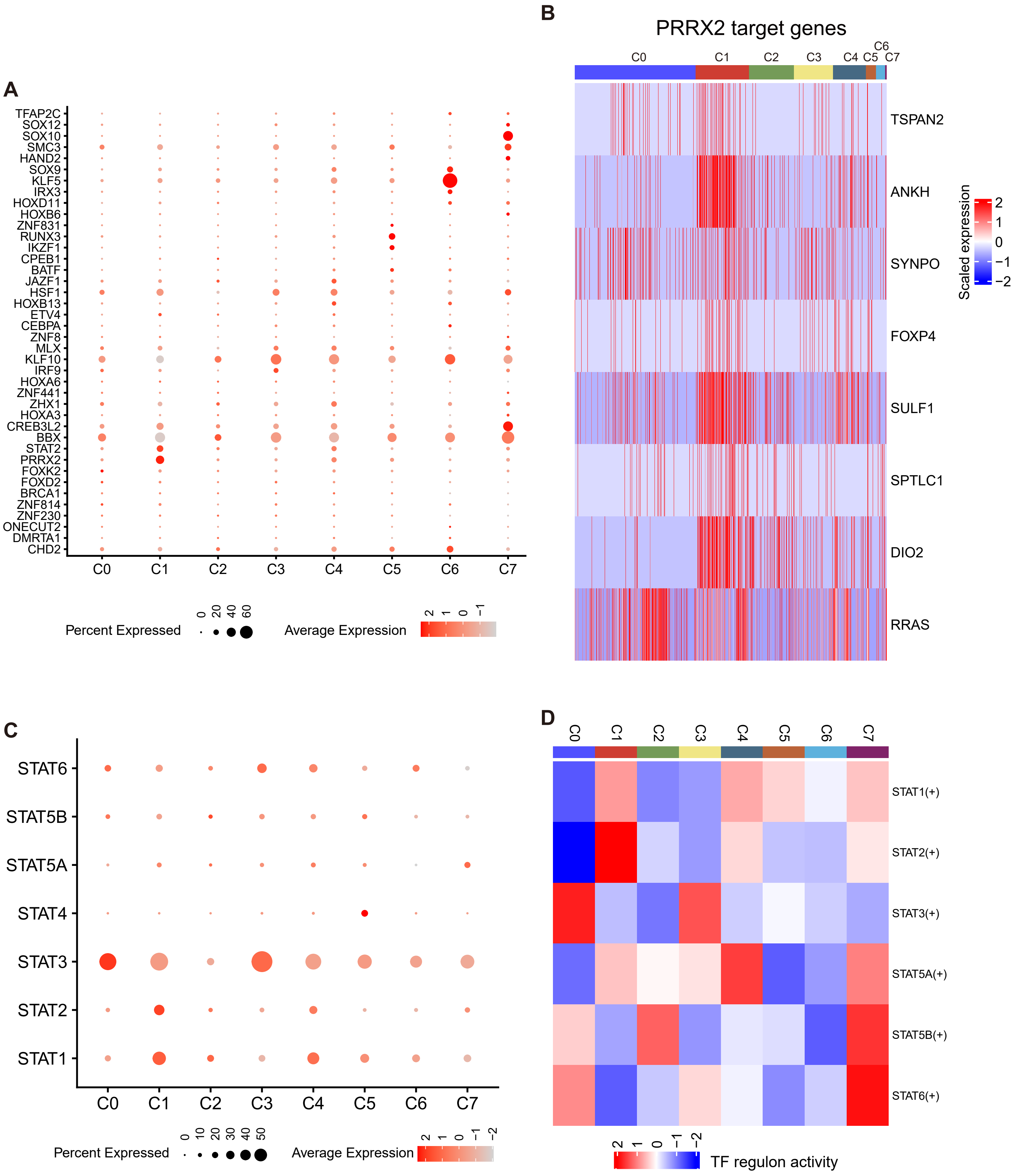

Supplemental figure 3. Relative expression of TFs in each annotated subpopulation of CAFs. A. Dot plot showing the expression levels of top 5 transcription factors predicted by pySCENIC for each CAFs subtype. **B.** Heatmap showing the expression levels of PRRX2 target genes in CAFs subtypes. **C.** Dot plot showing the expression levels of STAT family members for each CAFs subtype. **D.** Heatmap showing the regulon activities of STAT family members for each CAFs subtype predicted by pySCENIC.

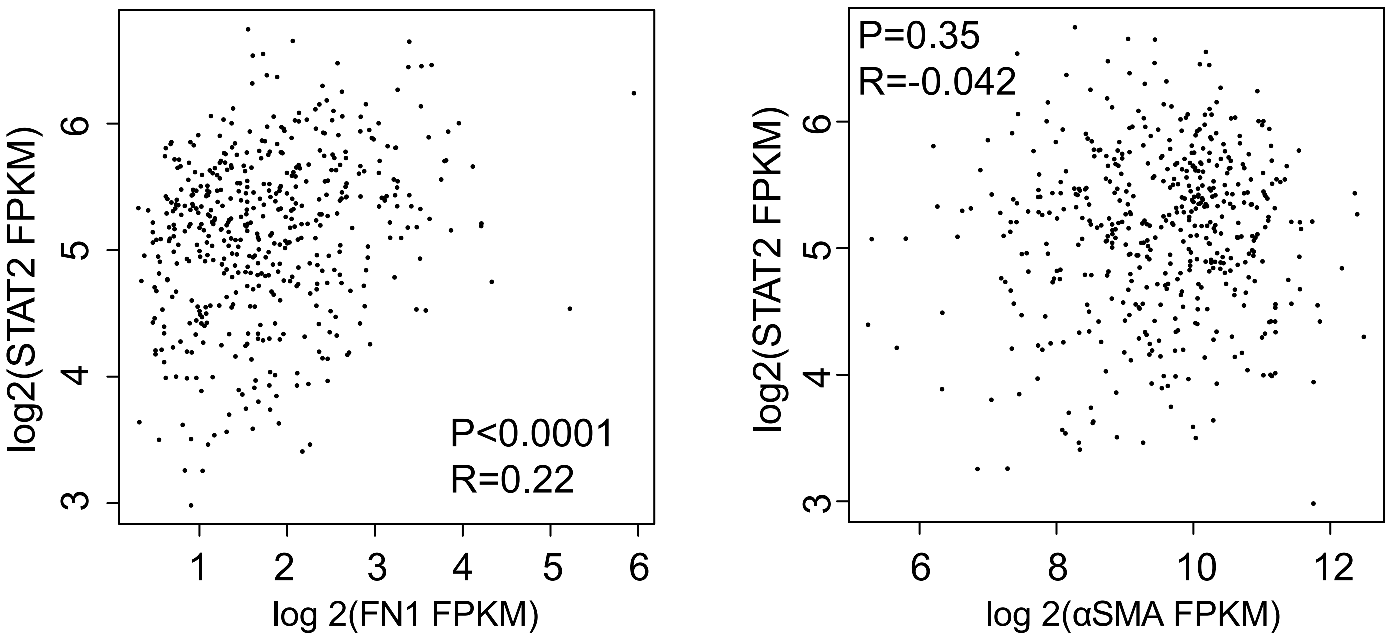

Supplemental figure 4. Expression correlation of STAT2 with CAFs-C1 marker (FN1) and CAFs-C0 marker (αSMA) in the TCGA prostate cancer patient cohort.

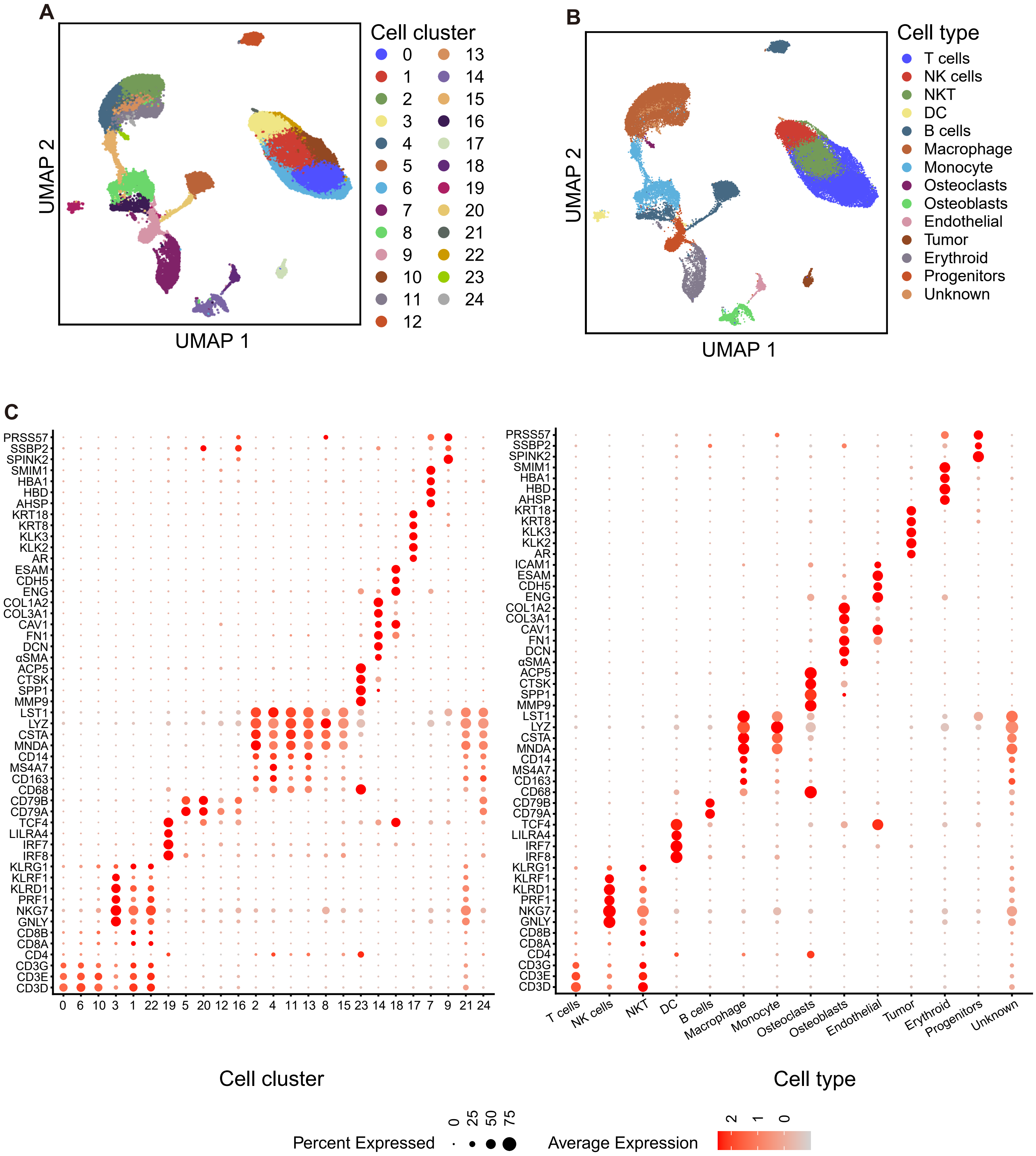

Supplemental figure 5. ScRNA-seq analysis of bone metastatic lesions. A. UMAP plot of single cells in 7 bone metastatic lesions. **B.** UMAP plot of cell types in 7 bone metastatic lesions. **C.** Dot plot showing the expression levels of cell type marker genes for each cell cluster (left) and each cell type (right).

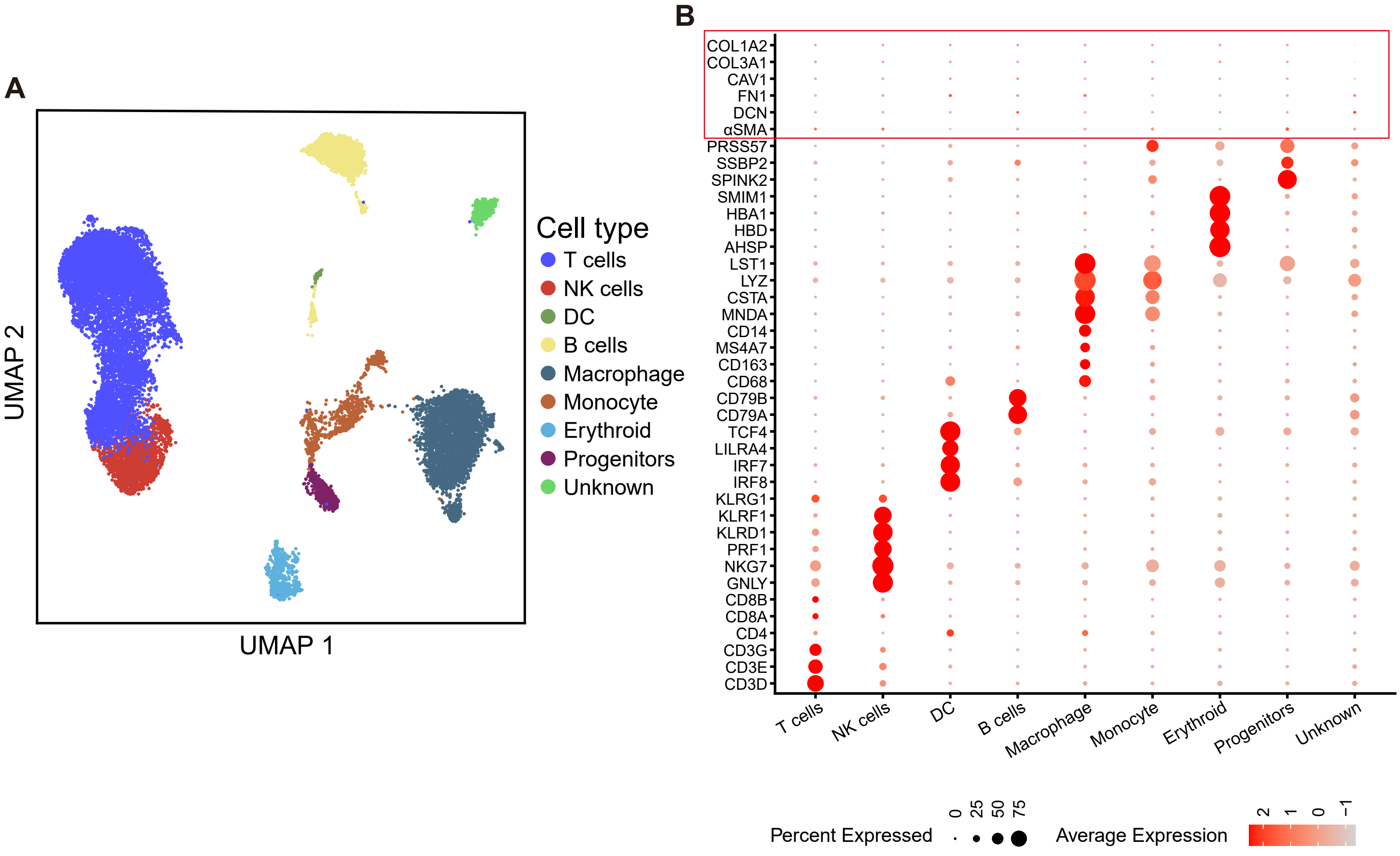

Supplemental figure 6. ScRNA-seq analysis of 8 normal bone tissues. A. UMAP plot of cell types in 8 normal bone tissues. **B.** Dot plot showing the expression levels of cell type marker genes for each cell type.

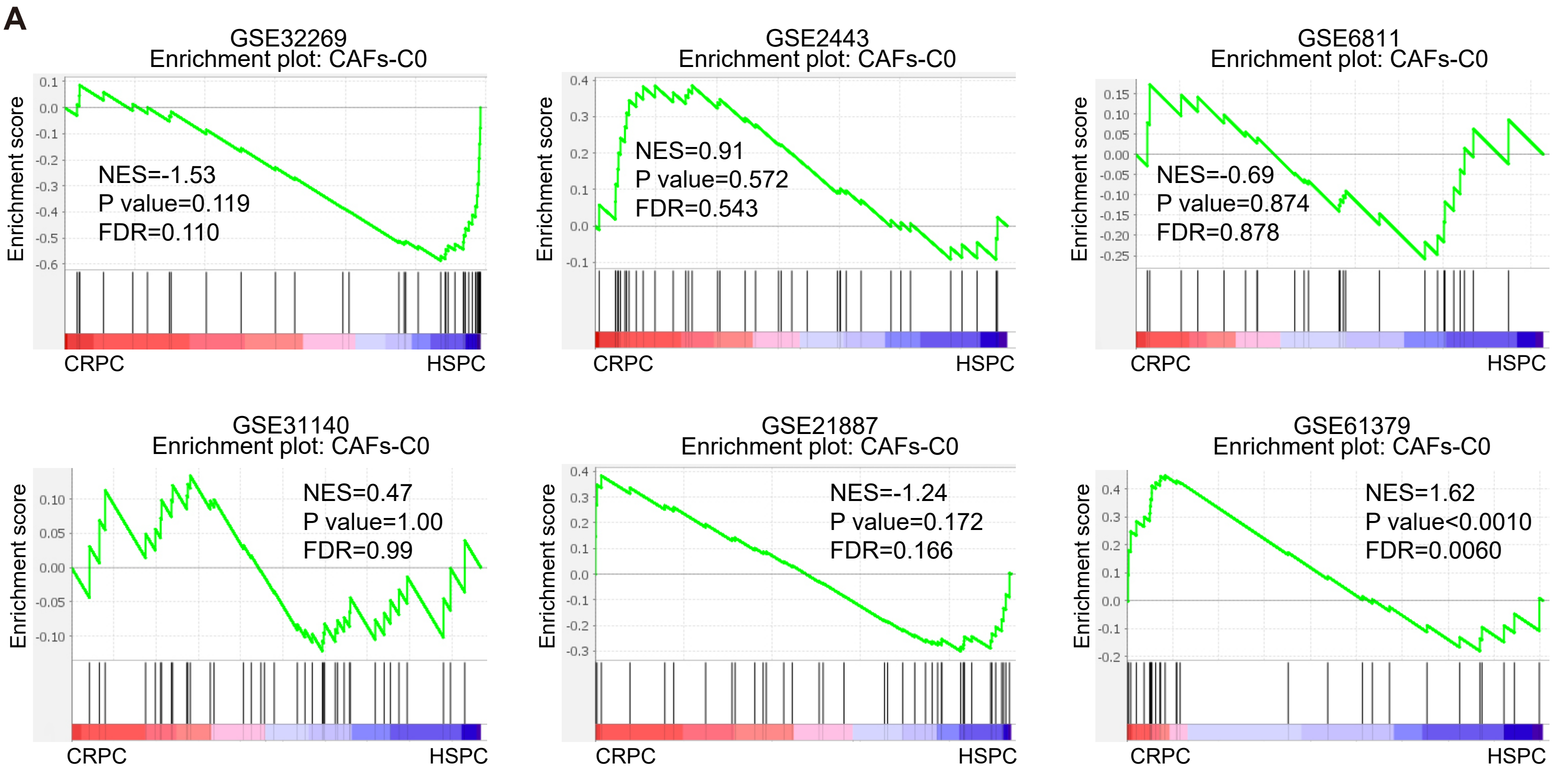

Supplemental figure 7. CAFs subtype signatures in bulk-seq profiles. A. GSEA analysis for CAFs-C0 signature between HSPC versus CRPC samples.

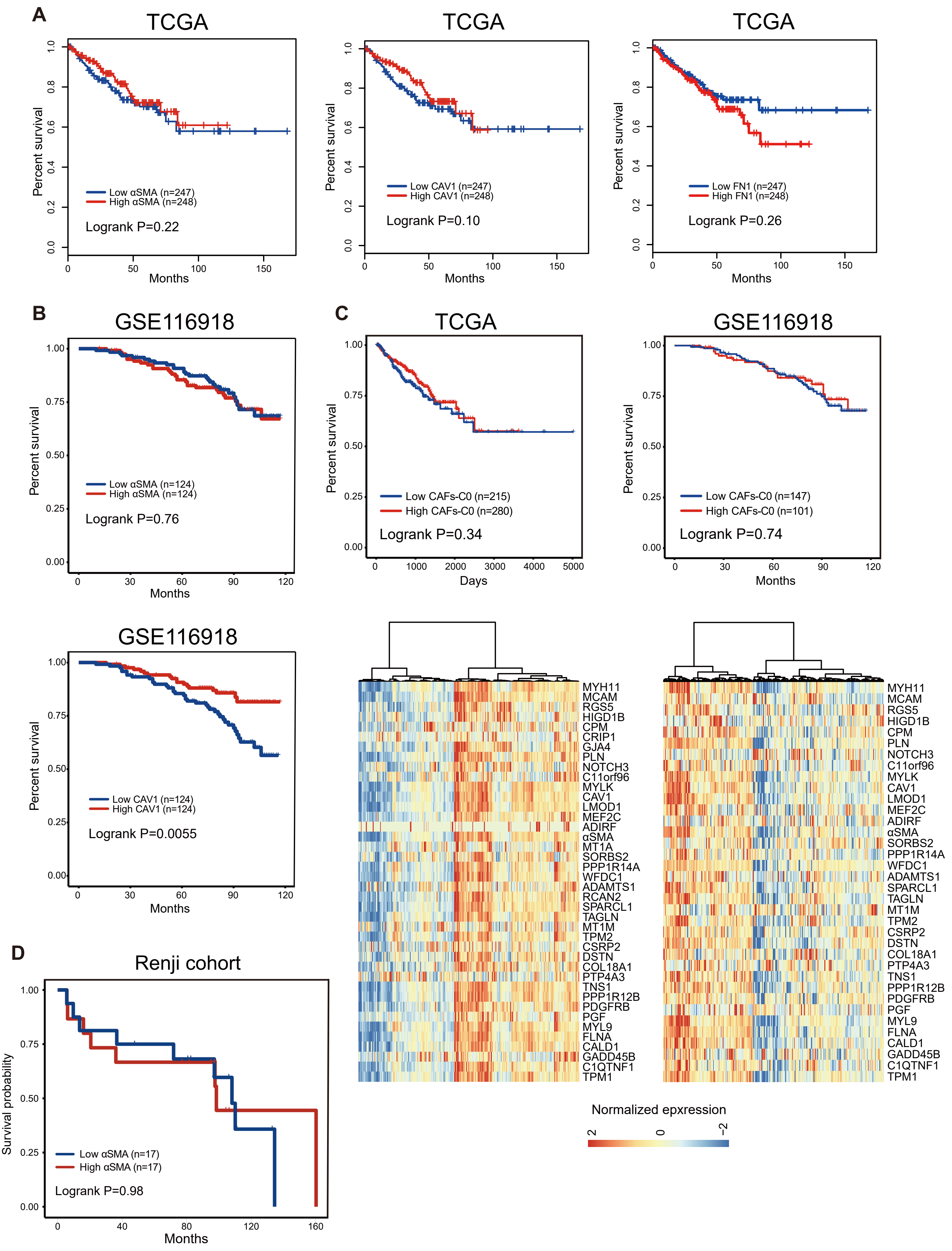

A
TCGA
Percent survival
Low αSMA (n=247)
High αSMA (n=248)
Logrank P=0.22
Months
TCGA
Percent survival
Low CAV1 (n=247)
High CAV1 (n=248)
Logrank P=0.10
Months
TCGA
Percent survival
Low FN1 (n=247)
High FN1 (n=248)
Logrank P=0.26
Months
B
GSE116918
Percent survival
Low αSMA (n=124)
High αSMA (n=124)
Logrank P=0.76
Months
C
TCGA
Percent survival
Low CAFs-C0 (n=215)
High CAFs-C0 (n=280)
Logrank P=0.34
Days
GSE116918
Percent survival
Low CAFs-C0 (n=147)
High CAFs-C0 (n=101)
Logrank P=0.74
Months
GSE116918
Percent survival
Low CAV1 (n=124)
High CAV1 (n=124)
Logrank P=0.0055
Months
MYH11
MCAM
RGS5
HIGD1B
CPM
CRIP1
GJA4
PLN
NOTCH3
C11orf96
MYLK
CAV1
LMOD1
MEF2C
ADIRF
αSMA
MT1A
SORBS2
PPP1R14A
WFDC1
ADAMTS1
RCAN2
SPARCL1
TAGLN
MT1M
TPM2
CSRP2
DSTN
COL18A1
PTP4A3
TNS1
PPP1R12B
PDGFRB
PGF
MYL9
FLNA
CALD1
GADD45B
C1QTNF1
TPM1
MYH11
MCAM
RGS5
HIGD1B
CPM
PLN
NOTCH3
C11orf96
MYLK
CAV1
LMOD1
MEF2C
ADIRF
αSMA
SORBS2
PPP1R14A
WFDC1
ADAMTS1
SPARCL1
TAGLN
MT1M
TPM2
CSRP2
DSTN
COL18A1
PTP4A3
TNS1
PPP1R12B
PDGFRB
PGF
MYL9
FLNA
CALD1
GADD45B
C1QTNF1
TPM1
Normalized epxression
2
0
-2
D
Renji cohort
Survival probability
Low αSMA (n=17)
High αSMA (n=17)
Logrank P=0.98
Months

Supplemental figure 8. Prognostic significance of CAFs-C0 signature in prostate cancer. A. The comparison of progression-free survival between patient subgroups divided by the expression levels of αSMA, CAV1, and FN1 in TCGA PRAD dataset. **B.** The comparison of biochemical recurrence-free survival between patient subgroups divided by the expression levels of αSMA and CAV1 in GSE116918 dataset. **C.** The comparison of biochemical recurrence-free survival between patient subgroups divided by the expression level of αSMA in Renji cohort. **D.** Bottom: Hierarchical clustering defined two CAFs-C0 subtypes based on the normalized expression profiles of 40 CAFs-C0 signature genes in TCGA and GSE116918 dataset. Top: Kaplan-Meier analysis showed CAFs-C0 signature was not associated with prognosis in TCGA-PRAD and GSE116918 dataset.
